# Strukturierte Patientenübergabe in Hochrisikobereichen: Evidenz und Empfehlungen für die praktische Implementierung

**DOI:** 10.1007/s00101-022-01249-x

**Published:** 2023-02-07

**Authors:** J. Fliegenschmidt, M. J. Merkel, V. von Dossow, B. Zwißler

**Affiliations:** 1 Institut für Anästhesiologie und Schmerztherapie, HDZ NRW, Ruhr-Universität Bochum, Georgstr. 11, 32545 Bad Oeynhausen, Deutschland; 2 Oregon Health & Science University, Mail Code: Mission Control UHS 9C40F, 3181 SW Sam Jackson Park Road, 97239 Portland, OR USA; 3 Institut für Anästhesiologie, Klinikum der Universität München, LMU München, 81377 München, Deutschland

**Keywords:** Strukturierte Übergaben, Kommunikationsstile, Hochrisikobereiche, Notfallmanagement, Hand-Over, Conmmunication skills, High-risk area, Emergency management

## Abstract

Die perioperative Medizin ist ein Hochrisikobereich, der besonders anfällig für Kommunikationsdefizite und -fehler ist. Das Schema „situation, background, assessment, recommendation“ (SBAR) bietet einen einfach anzuwendenden Kommunikationsleitfaden, der mit einer verbesserten Qualität der Übergabe assoziiert ist. Im März 2022 ist die Verwendung des SBAR-Schemas in der Perioperativmedizin durch die DGAI schon in zweiter Auflage empfohlen worden. Darüber hinaus hat die moderne Kommunikationsforschung ein ganzes Bündel von Maßnahmen identifiziert, die essenzielle Voraussetzungen für eine effektive Teamarbeit und die Gewährleistung der Patientensicherheit schaffen. Das SBAR-Schema ist eine Möglichkeit, strukturierte Kommunikation im klinischen Alltag umzusetzen. Entscheidend sind die konsequente Nutzung und eine klare Definition der Handlungsabläufe. Nur so können Kommunikationsdefizite in Hochrisikobereichen schneller identifiziert und durch Einführung eines strukturierten Übergabekonzeptes reduziert werden. Unabdingbar bleibt das gemeinsame Verständnis für die Notwendigkeit, diese Konzepte zu erlernen, umzusetzen und als Team zu trainieren. Das übergeordnete Ziel einer Kultur der Patientensicherheit ist nur durch die konsequente Zusammenarbeit des interprofessionellen Teams und durch das Vorleben der Führungskräfte erreichbar.

## Abstract (en)

The perioperative setting is a high-risk environment which is particularly susceptible to communication deficits and errors. The situation, background, assessment, recommendation (SBAR) approach provides an intuitive guideline for team communication, which is associated with an improved quality of the handover. The German Society for Anaesthesiology and Intensive Care Medicine (DGAI) has updated its recommendations in March 2022 and continues to endorse the use of the SBAR template. The impact of tools used for structured communication during patient handover are often studied in the context of a larger bundle of measures. The SBAR template is one option for establishing structured communication in clinical practice. Successful implementation is supported by clearly defined standard workflows to promote consistent use. This standardization identifies common communication barriers and assists in resolving them in a high-risk environment. A common understanding of the inherent values, and a shared interest in learning, applying, and training these techniques are paramount in establishing a culture of patient safety. This can only be reached through excellent interprofessional teamwork and supportive leadership.

„Wir tendieren dazu, Ideen für Tatsachen zu halten, was Chaos in der Welt schafft.“ (Paul Watzlawick, Kommunikationswissenschaftler)

Im März 2022 hat die Deutsche Gesellschaft für Anästhesiologie und Intensivmedizin (DGAI) ihre erstmals 2016 veröffentlichte Empfehlung zur Verwendung des SBAR-Schemas für die strukturierte Patientenübergabe in Risikobereichen aktualisiert. Das SBAR(„Situation, Background, Assessment and Recommendation“)-Schema ist ein zentraler Baustein, dessen Wert sich aber auch in Implementierungsprozessen niederschlägt. Eine engagierte Umsetzung durch die Teams der direkten Patientenversorgung ist der Schlüssel zu erfolgreicher und gewinnbringender Nutzung.

## Kommunikation im Team

Medizin ist Teamarbeit. Das wird im perioperativen und im intensivmedizinischen Bereich besonders evident. Elementarer Bestandteil einer Tätigkeit in Risikobereichen ist es daher, alle Beteiligten durch gewissenhafte Dokumentation und strukturierte Übergaben auf einen Informationsstand zu bringen, der einen sicheren Beitrag zur Behandlung ermöglicht.

Es ist belegt, dass ein strukturierter Austausch von Informationen in dieser Konstellation einen besonderen Stellenwert besitzt. Patient:innenübergaben in Bereichen der intensivmedizinischen und perioperativen Versorgung sind vulnerabel gegenüber Störeinflüssen, welche in nennenswertem Maße auch organisatorischen Umständen zuzuschreiben sind [10, 19, 20]. Ein ungenügender Austausch von Informationen kann dabei direkt zu Behandlungsfehlern führen, die auch rechtliche Konsequenzen nach sich ziehen können [9, 14]. Außerdem resultiert eine schlechtere Zusammenarbeit im interdisziplinären Team, was einen negativen Einfluss auf die gemeinsame Leistung und gegenseitige Wertschätzung hat [18].

Konsequenterweise wird die strukturierte Übergabe anhand des SBAR-Schemas von vielen Fachgesellschaften empfohlen, darunter die Weltgesundheitsorganisation (WHO) und die Deutsche Gesellschaft für Anästhesiologie und Intensivmedizin (DGAI) [4, 7]. Die Effekte einer solchen Maßnahme sind dabei schwierig in Gänze zu erfassen. Es geht nicht nur um eine buchstabengetreue Befolgung einer Übergabe, sondern vielmehr, dass die Informationen vom Sender zum Empfänger konsitent transportiert und inhaltlich verstanden werden. *Ziel der Übergabe ist nicht, die eigene Verantwortung abgegeben zu haben; die strukturierte Übergabe soll vielmehr das übernehmende Behandlungsteam in die Lage versetzen, Zuständigkeit und Verantwortung für die weitere Versorgung zu übernehmen. *Übergeordnetes Ziel ist eine Kultur der Patientensicherheit, in der sinnvolle Redundanzen geschaffen werden. Dadurch tragen alle Versorgenden kollektiv zur Fehlervermeidung bei.

Dass es diesbezüglich noch Entwicklungspotenzial gibt, suggerieren aktuell veröffentlichte Daten von Marcus et al. [11]. Bezüglich SBAR berichten die Autoren, dass zum Zeitpunkt der Umfrage (2017) 86 % der Teilnehmer:innen eine Notwendigkeit sahen, den Übergabeprozess durch Hilfsmittel zu verbessern. Das SBAR-Schema jedoch war nur ca. 30 % der Befragten bekannt; eine regelmäßige Anwendung berichteten nur 8 %. Weiterhin heben die Autoren eine gravierende Diskrepanz in der Wahrnehmung der Kommunikations- und Fehlerkultur hervor: Konstruktive Besprechung fehlerhafter Verläufe berichteten Chef‑/Oberärzt:innen deutlich häufiger als Fach‑/Assistenzärzt:innen; ein Bloßstellen von Kolleg:innen berichteten Erstere deutlich seltener als Letztere. In einer alarmierenden Konsequenz gaben mehr als ein Viertel der befragten Fach- und Assistenzärzt:innen an, Fehler lieber zu verschweigen, ebenso mehr als jede:r zehnte der Chef- sowie knapp jede:r fünfte der Oberärzt:innen. Die Daten wurden unter ärztlichem Personal erhoben; mögliche interdisziplinäre Differenzen bleiben unbeleuchtet. Insgesamt ergibt sich das Bild einer eher restriktiven Kommunikationskultur, die den oben formulierten Zielen entgegensteht.

## Den Übergabeprozess gestalten

Die Einführung einer substanziellen Veränderung bedarf gewisser Vorbereitung, z. B. im Sinne eines Veränderungsmanagements. Dabei sollten die betroffenen Abteilungen ebenso einbezogen werden wie diejenigen, deren Arbeitsweise standardisiert wird. Für eine erfolgreiche Umsetzung braucht es Führungskräfte, die hinter der Prozessänderung stehen, und möglichst breite Unterstützung in der gesamten Belegschaft. Dafür sollten die Beweggründe hinter der Veränderung sichtbar gemacht und Rückmeldungen adressiert werden.

Eine sinnvolle und umfassende Implementierung des strukturierten Übergabeprozesses ist aber mit der Vorgabe des SBAR-Schemas nicht abgeschlossen. Idealerweise wird diese zusammen mit einer Standardarbeitsanweisung (SAA) eingeführt, die den Übergabeprozess beschreibt und im täglichen Arbeitsablauf verankert. Diese sollte in Fortbildungen vorgestellt und motiviert werden. Interdisziplinäres Training verstärkt die positiven Effekte, ist jedoch im klinischen Alltag nicht überall möglich. Das sollte aber nicht entmutigen, den Prozess zu beginnen (→ 5).

**Figure 1:**
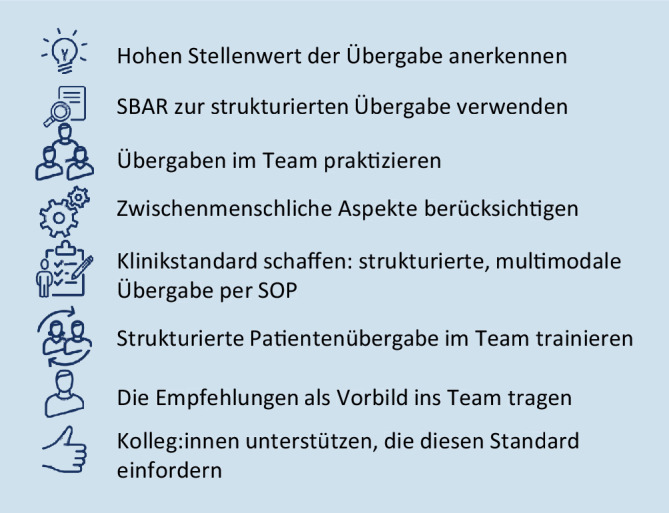

Die folgende Aufzählung gibt einen Anhalt dafür, welche Überlegungen einer Reform zugrunde liegen sollten (Abb. 1):

1. *Die Übergabe ist ein obligatorischer und sicherheitsrelevanter Prozess der täglichen Arbeit*. Als sicherheitsrelevanter Prozess sollte die Übergabe standardisiert und trainiert werden. Als Bestandteil der täglichen Arbeit sollte die strukturierte Patientenübergabe obligat stattfinden.
2. *Die Zuhilfenahme von SBAR als einheitliches Übergabeschema verbessert Informationsgehalt und Rezeption der Übergabe. *Die Vorteile eines „mnemonic“-gestützten Übergabeschemas sind in Studien vielfach nachgewiesen. Das SBAR-Schema wird dazu national und international empfohlen.
3. *Die Übergabe sollte in Anwesenheit und unter Einbeziehung aller Beteiligten vollzogen werden*. Das Einbeziehen aller Teammitglieder schafft Sicherheit im Sinne einer informationellen Redundanz, bereichert aber auch die Übergabe um die Perspektive der verschiedenen Professionen und stärkt die interprofessionelle Zusammenarbeit.
4. *Die Übergabe sollte in einer wertschätzenden Atmosphäre stattfinden*. Kommunikation ist immer auch von zwischenmenschlichen Faktoren geprägt. Der fachliche Austausch gelingt dann besonders gut, wenn die Beteiligten sich nicht um persönliche Kritik oder Bloßstellung sorgen müssen. Inhalte sollten fachlich diskutiert und Beiträge grundsätzlich willkommen geheißen werden.
5. *Die Übergabe sollte mit möglichst wenig Ablenkung stattfinden*. Maßnahmen an Patient:innen sollten nur dann parallel durchgeführt werden, wenn ein Aufschieben eine unmittelbare Gefährdung bedeutet.
6. *Die Übergabe sollte multimodal stattfinden und kann durch digitale Technologien gestützt werden*. Die schriftliche Vorbereitung in Adhärenz zum gegebenen Schema hilft, die relevanten Punkte in der Übergabesituation schemagerecht zu erinnern. Eine Dokumentation kann auch nach der Übergabe noch konsultiert werden; die digitale Umsetzung ermöglicht sogar die vorbereitende Übersendung.
7. *Die Übergabe kritisch kranker Patienten sollte vorangemeldet erfolgen*. Zur Übernahme insbesondere kritisch erkrankter Patienten müssen vom übernehmenden Team umfangreiche Vorbereitungen getroffen werden. Eine beispielsweise telefonische Voranmeldung ermöglicht die Übergabe an ein vorbereitetes Team, sodass die Ablenkungen in der Übergabesituation möglichst reduziert sind.
8. *Die strukturierte Patientenübergabe sollte als Teil der interprofessionellen Zusammenarbeit in Studium und Ausbildung gelehrt und in regelmäßigen Trainings vertieft werden*. Das regelmäßige Beüben kollaborativer Situationen verbessert die Teamleistung, stärkt das Team und macht die erarbeiteten Kompetenzen besser abrufbar.

Möglicherweise gibt es noch weitere Arbeitsschritte im postoperativen Management, die die Übergabe beeinflussen können. Vordefinierte Versorgungsstufen zur telefonischen Anmeldung beispielsweise können den zu erwartenden Ressourcenbedarf vermitteln. Es wird dadurch verhindert, dass eine telefonische Vorabübergabe, die nicht in einem interdisziplinären Plenum stattfindet, die eigentliche Übergabe am Bett beeinträchtigt oder gar ersetzt.

## Das SBAR-Schema

Das am häufigsten gebrauchte und durch die DGAI empfohlene Schema für eine strukturierte Übergabe ist das sog. SBAR-Schema [4, 5, 7, 13, 15, 19]. Es gibt die Einteilung von Informationen in folgende Kategorien vor:

- *S* – „Situation“ (Situation)
  Die aktuellen Umstände werden in Kürze umrissen. Name, Alter, Geschlecht und der aktuelle Zustand (führende Diagnose, Zustand nach Trauma oder Intervention) werden benannt. Insbesondere der Hinweis auf einen derzeit kritischen Patientenzustand sollte hier erfolgen.
- *B* – „Background“ (Hintergrund)
  Eine fokussierte Patientenanamnese mit relevanten Vorerkrankungen, Medikation und dem aktuellen Verlauf. Risikofaktoren und Allergien sowie biografische Umstände, die in der Versorgung besondere Berücksichtigung finden sollen, werden ebenfalls übergeben.
- *A* – „Assessment“ (Zustand)
  Eine zielgerichtete Darstellung der selbst erhobenen, objektiven Untersuchungsbefunde. Hier soll ein möglichst genaues Bild des aktuellen Gesundheits- und Versorgungsstatus wiedergegeben werden.
- *R* – „Recommendation“ (Empfehlung)
  Die geplante weitere Versorgung soll hier skizziert und mögliche Komplikationen sollen explizit angesprochen werden. Insbesondere ist hier Platz für subjektive Einschätzungen, auch über den erwarteten Verlauf.

Die Empfehlung der DGAI enthält eine beispielhafte Ausgestaltung für die postoperative Übergabe.

Ein wichtiger Aspekt dieser Strukturierung ist die Trennung von objektiven und subjektiven Inhalten. Die Übergabe gibt dem weiterbehandelnden Team damit ein Maximum an verwertbaren Informationen. Die klare Trennung hilft dabei, Informationen richtig einzuordnen und gezielt nachzufragen, wenn die eigene Einschätzung der objektiven Befunde nicht kongruiert. Das fördert den professionellen Dialog und verhindert Missverständnisse.

Wenn Sie nach Operation einen Patienten „mit einem arteriellen Mitteldruck von 65 mm Hg und niedrigdosierten Katecholaminen“ übergeben, weil eine Stabilisierung wider Erwarten ohne Katecholamine nicht erreichbar war, dann haben Sie eine andere Vorstellung vom Patientenzustand als ihr Gegenüber, das interpretiert, dass der Patient bereits aggressiv von Katecholaminen entwöhnt worden ist und nun mit einem Restbedarf an ihn übergeben wird. Die subjektive kontextuelle Einordnung (unerwartet noch katecholaminpflichtig vs. bald nicht mehr katecholaminpflichtig) ergänzt die Daten um Hintergrundwissen.

### Identifikation: das ISBAR-Schema

Eine Abwandlung des SBAR-Schemas erweitert das Mnemonic an erster Stelle um ein I für „identification“ [3]. Hier soll den medizinischen Inhalten die Identifikation von Patient:in und Teams vorangestellt werden. Die Teams stellen sich gegenseitig mit Rollen und Namen vor und schaffen damit die zwischenmenschliche Grundlage für gute Kommunikation. Sind die Kolleg:innen bekannt, bietet es sich v. a. an, eine:n Teamleader:in zu identifizieren. Bei Ad-hoc-Teams allerdings, wie sie in manchen Bereichen strukturell unumgänglich sind, aber auch durch hohe Personalfluktuation zustande kommen, ist die gegenseitige Vorstellung ein zentraler Bestandteil.

## Handlungsempfehlungen

- Für alle an der Übergabesituation Beteiligten
  - Eine Sicherheitskultur wird nicht mit Dienstanweisungen oder SAA etabliert; kulturellen Wandel müssen alle Beteiligten mittragen:
  - Achten Sie auf die Einhaltung der Standards.
  - Wenden Sie die Elemente, die in Ihrem Einflussbereich liegen, konsequent an.
  - Wenn Sie Bemerkungen oder Rückfragen haben, bringen Sie sie auch unaufgefordert an. Unterstützen Sie Kolleg:innen, die Kommunikation auf diesem Wege einfordern.
  - Es liegt an jedem/jeder Einzelnen, Respekt und Wertschätzung für die Arbeit aller Beteiligten zu signalisieren.
  - Wenn Sie moderierenden Einfluss nehmen müssen, tun Sie das ohne persönlichen Bezug. Für persönliche Kritik ist das Plenum nicht der richtige Ort.
- Auszubildende und Studierende
  - Nutzen Sie die Gelegenheiten, die sich Ihnen bieten, unterschiedliche Arbeitsweisen kennenzulernen.
  - Reflektieren Sie regelmäßig das Verhalten Ihrer Fachkollegen. Vergleichen Sie unterschiedliche Übergabesituationen und gleichen Sie sie mit den vorliegenden Empfehlungen ab.
  - Wenn Sie selbst eine Patientenübergabe machen, verwenden Sie das SBAR-Schema.
- Ärzt:innen ohne leitende Funktion
  - Machen Sie sich der Vorbildfunktion Ihrer Rolle bewusst. Wenn Sie selbst die Patientenübergabe machen, verwenden Sie ein Schema zur strukturierten Übergabe.
  - Begrüßen Sie konstruktive Beiträge und zweckdienliche Nachfragen, unabhängig davon, wer sie vorbringt.
  - Nutzen Sie als Empfänger die Ihnen vertraute Struktur, um die erhaltenen Informationen zusammenzufassen.
  - Sprechen Sie über die Bedeutung guter Kommunikation und wertschätzender Zusammenarbeit im Team. Geben Sie die Empfehlung der Fachgesellschaft weiter.
- Leitende Ärzt:innen, Abteilungs- und Klinikleitung
  - Teilen Sie als Führungspersönlichkeit Ihre Ziele und Visionen mit Ihren Mitarbeitern. Schaffen Sie ein gemeinsames Verständnis für den weitreichenden Einfluss Ihrer Maßnahmen.
  - Erarbeiten Sie SAA für den strukturierten Übergabeprozess. Werben Sie für ein gemeinsames Vorgehen aller betroffenen Abteilungen.
  - Kommunizieren Sie die Empfehlung der Fachgesellschaft und setzen Sie Ihre SAA konsequent um. Eine Sicherheitskultur muss aktiv entwickelt und gelebt werden; die Anordnung per Dienstanweisung ist nicht erfolgversprechend.

## Wissenschaftliche Hintergründe: SBAR

Das SBAR-Schema ist international erprobt, und positive Effekte auf interdisziplinäre Zusammenarbeit und Kommunikation sowie auf die Sicherheitskultur sind belegt [4, 5, 25]. Auch die ISBAR-Variante des Schemas wird von mehreren Autor:innen empfohlen [3, 6, 12]. Die Einführung strukturierter Übergabeprotokolle und Anwendung von Mnemonics zur Kommunikation wird in der Literatur häufig als Maßnahmenbündel studiert: Weinger et al. zeigten anhand der Einführung eines strukturierten Übergabeprotokolls im Aufwachraum durch multimodale Schulungen eine deutliche Verbesserung in der Qualität der Übergabe über einen Zeitraum von ca. anderthalb Jahren [26]. Auch die Einführung eines „I-PASS Handoff Bundle“ als Alternative zu SBAR war sowohl mit einer signifikanten Reduktion medizinischer Fehler (23 %) und vermeidbarer Ereignisse (30 %) als auch verbesserter Kommunikation assoziiert, ohne negativen Effekt auf die Arbeitsabläufe [24]. In einem prospektiven Vergleich zwischen 2 Krankenhäusern fanden Raandma et al. [19] signifikante Verbesserungen in Bezug auf gemeldete Kommunikationsfehler, (11 % vs. 31 %), die Genauigkeit der Kommunikation sowie des „Sicherheitsklimas“. Dafür wurden im Interventionsklinikum Schulungen für das gesamte interdisziplinäre Team durchgeführt, Taschenkarten mit der Merkhilfe verteilt und das SBAR-Schema an strategischen Punkten plakatiert. Auch De Meester et al. [13] stellten signifikante Verbesserungen der Kommunikation nach Einführung des SBAR-Schemas fest: Ein signifikanter Anstieg von Meldungen über Zustandsverschlechterungen (4 % vs. 35 %) und ungeplante Aufnahmen auf die Intensivstation (0,34 ‰ vs. 0,99 ‰) schlug sich in einer ebenfalls signifikanten Reduktion unerwarteter Todesfälle (RRR: −227 %; 95 %-KI [−793; −20]; *p* < 0,001) nieder. Sie konnten damit eine Relevanz für das Patienten-Outcome zeigen. Die Autoren erklären die Veränderung mit einer erhöhten Aufmerksamkeit durch besseren Informationsaustausch, der u. a. auf eine verbesserte interprofessionelle Kommunikation zurückgeführt wird. Das ist vereinbar mit Untersuchungen, die in strukturierter Kommunikation eine Chance sehen, hierarchische Barrieren zu egalisieren [23].

Insgesamt ist also grundsätzlich eine deutliche kommunikative Verbesserung bezüglich der Übergabe zu erreichen, und es gibt gleich mehrere geeignete Konzepte. Kliniken, in denen knappe Ressourcen einer Umsetzung komplexer Maßnahmenbündel im Wege stehen, sollten deshalb nicht grundsätzlich von dem Vorhaben absehen [16, 17]. Veränderungen können auch sequenziell umgesetzt werden, beispielsweise sind die Einführung eines Übergabeschemas, die Bereinigung der Übergabesituation von Ablenkungen und Zeitdruck [2, 22] und die Verwendung einer Kombination aus schriftlicher und mündlicher Übergabe [8] effektive Einzelmaßnahmen.

Letztlich bleibt aber unklar, welche Einflüsse konkret für die Verbesserung der Kommunikation verantwortlich sind. Riesenberg et al. konnten 2009 auf Basis von 46 Publikationen insgesamt 24 verschiedene Übergabe-Mnemonics identifizieren [21]. Zwar wurde das SBAR-Schema am häufigsten studiert, eine Überlegenheit gegenüber anderen Schemata ist jedoch nicht belegt. Insbesondere in den Studien mit umfangreichen Maßnahmenbündeln und Trainings bleibt die Frage offen, welche Einzelmaßnahmen tatsächlich effektiv sind [19, 24, 26]. Insgesamt kommen aktuelle Arbeiten zu dem Ergebnis, dass die Heterogenität und Qualität der verfügbaren Studien die Zusammenführung in eine hochwertige Metaanalyse derzeit nicht zulassen [1, 15].

## Fazit für die Praxis

- Die Übergabe nach SBAR ist eine evidenzbasierte Intervention, die als Teil eines Maßnahmenbündels die Patientenversorgung verbessert.
- Kürzlich veröffentlichte Daten lassen einen Bedarf an solchen Maßnahmen deutlich erkennen.
- Ein Maßnahmenbündel muss nicht zwangsläufig en bloc etabliert werden.
- Der Erfolg ist von der Unterstützung in der Belegschaft abhängig; auch unter den idealen Bedingungen muss die strukturierte Patientenübergabe letztlich von den Behandelnden in den entsprechenden Hochrisikobereichen aktiv umgesetzt werden.
- Die Umsetzung fördert die konstruktive interdisziplinäre Zusammenarbeit.
